# Brain activity underlying negative self- and other-perception in adolescents: The role of attachment-derived self-representations

**DOI:** 10.3758/s13415-017-0497-9

**Published:** 2017-02-06

**Authors:** Martin Debbané, Deborah Badoud, David Sander, Stephan Eliez, Patrick Luyten, Pascal Vrtička

**Affiliations:** 1 Developmental Clinical Psychology Research Unit, Faculty of Psychology and Educational Sciences, University of Geneva, Geneva, Switzerland; 2 Office Médico-Pédagogique Research Unit, Department of Psychiatry, University of Geneva School of Medicine, Geneva, Switzerland; 3 Research Department of Clinical, Educational and Health Psychology, University College London, London, UK; 4 Swiss Center for Affective Sciences, University of Geneva, Geneva, Switzerland; 5 Laboratory for the Study of Emotion Elicitation and Expression, Department of Psychology, University of Geneva, Geneva, Switzerland; 6 Faculty of Psychology and Educational Sciences, University of Leuven, Leuven, Belgium; 7 Department of Social Neuroscience, Max Planck Institute for Human Cognitive and Brain Sciences, Leipzig, Germany

**Keywords:** Self- versus other-processing, Adolescence, Attachment theory, Negative self-model, fMRI

## Abstract

One of teenagers’ key developmental tasks is to engage in new and meaningful relationships with peers and adults outside the family context. Attachment-derived expectations about the self and others in terms of internal attachment working models have the potential to shape such social reorientation processes critically and thereby influence adolescents’ social-emotional development and social integration. Because the neural underpinnings of this developmental task remain largely unknown, we sought to investigate them by functional magnetic resonance imaging. We asked n = 44 adolescents (ages 12.01–18.84 years) to evaluate positive and negative adjectives regarding either themselves or a close other during an adapted version of the well-established self-other trait-evaluation task. As measures of attachment, we obtained scores reflecting participants’ positive versus negative attachment-derived self- and other-models by means of the Relationship Questionnaire. We controlled for possible confounding factors by also obtaining scores reflecting internalizing/externalizing problems, schizotypy, and borderline symptomatology. Our results revealed that participants with a more negative attachment-derived self-model showed increased brain activity during positive and negative adjective evaluation regarding the self, but decreased brain activity during negative adjective evaluation regarding a close other, in bilateral amygdala/parahippocampus, bilateral anterior temporal pole/anterior superior temporal gyrus, and left dorsolateral prefrontal cortex. These findings suggest that a low positivity of the self-concept characteristic for the attachment anxiety dimension may influence neural information processing, but in opposite directions when it comes to self- versus (close) other-representations. We discuss our results in the framework of attachment theory and regarding their implications especially for adolescent social-emotional development and social integration.

**Electronic supplementary material:**

The online version of this article (doi:10.3758/s13415-017-0497-9) contains supplementary material, which is available to authorized users.

## Introduction

### Attachment theory

Attachment theory, developed by John Bowlby and Mary Ainsworth (Ainsworth, Blehar, Walters, & Wall, 1978; Bowlby, 1969, 1980), postulates that humans are born with an innate attachment system. The biological function of this attachment system is to promote proximity seeking, particularly in times of stress and need; its primary aim is to enhance the chances of offspring survival and thus reproductive success. It is believed that all children become attached to their caregiver(s), also referred as to their primary attachment figure(s). Importantly, however, child-caregiver attachment can vary significantly in its underlying qualities. Through repeated early interactions with available and responsive attachment figures, children can develop a secure attachment. Conversely, if early interactions occur with unavailable, unresponsive, and/or inconsistent attachment figures, children are likely to develop an insecure attachment. In the course of development, the fundamental qualities of secure or insecure attachment are thought to become cognitively encoded by means of different internal self- and other-representations (Bartholomew & Horowitz, 1991), also referred to as internal working models (IWMs; Mikulincer & Shaver, 2007). Once established, these IWMs are believed to remain relatively stable throughout the lifespan and to considerably influence the perception of new significant others entering individuals’ lives. IWMs can therefore influence many social interactions with, in particular, friends, peers, romantic partners, and, eventually, a person’s own children, thereby completing the cycle of intergenerational attachment style transmission (Belsky, 2006; Mikulincer & Shaver, 2007; Pascuzzo, Cyr, & Moss, 2013; Shah, Fonagy, & Strathearn, 2010).

IWMs of self and others represent general expectations about the worthiness of the self and the availability of others (i.e., how do I think about my own and others’ value?) (Bartholomew & Horowitz, 1991; Griffin & Bartholomew, 1994). The self-model indicates the degree to which a person has internalized a sense of his/her own self-worth and therefore expects that others will respond positively during social interactions (i.e., positivity of self-concept). Consequently, the self-model is inversely associated with the degree of anxiety and dependency experienced in social relationships (generally mapping to the attachment anxiety dimension). In turn, the other-model indicates the degree to which others are expected to be generally available and supportive, and therefore corresponds to the tendency of seeking out or avoiding closeness in relationships (i.e., positivity of interpersonal relations, generally inversely mapping to the attachment avoidance dimension) (Bartholomew & Horowitz, 1991; Griffin & Bartholomew, 1994). These self- and other-models may be particularly important during adolescence, because one of the key developmental tasks of this period consists of engaging in new and meaningful relationships with peers and adults outside the family context.

### Attachment in adolescence

From an attachment theory perspective, adolescence is marked by teenagers’ separation from their family: usually, the amount of time (waking hours) spent with parents drops by an average of 20%. This reduction in time is related to the fact that new social roles (e.g., working, peer, and romantic relationships) are opening up for adolescents, and these roles take adolescents further away from their families (Moretti & Peled, 2004). Consequently, adolescents enter a new social-emotional phase of their life, a transition that requires them to integrate new and diverse experiences in relation to the world and oneself. Attachment-derived IWMs of self and others may critically shape and guide the social adaptation processes occurring during adolescence, and therefore significantly influence adolescents’ social-emotional developmental course and social integration.

Moretti and Peled (2004) provide a good, comprehensive review on how secure attachment during adolescence may support healthy development in normative samples. Their summarized findings indicate that secure (vs. insecure) adolescents enjoy more positive relationships and experience less conflict with family and peers, and that secure attachment during adolescence may be linked to a significant gain in social skills from age 16–18 years. Furthermore, Moretti and Peled (2004) show that secure attachment during adolescence is associated with fewer mental health problems and conduct problems in general, and fewer weight-related concerns and less frequent eating disorders in females specifically. These data therefore suggest that secure attachment during adolescence may beneficially affect personal development in terms of both a more positive self-representation (i.e., stronger positivity of the self-concept) and a more positive model of others (i.e., stronger positivity of interpersonal relations).

Despite the existing evidence of IWMs of self and others contributing to adolescent social-emotional development in important ways, very little is known about the putative underlying neural substrates.

### Attachment and brain imaging

Internal self- and other-representations underlying IWMs are considered central to attachment theory. However, to the best of our knowledge, no functional magnetic resonance imaging (fMRI) study has yet targeted their possible underlying neural substrates, particularly in adolescents.

Only one study to date has specifically investigated brain activation patterns in adolescents as a function of attachment (Vrtička et al., 2014). The main focus of that study was to elucidate the association of attachment styles (i.e., attachment avoidance vs. anxiety) with “social feedback processing,” which the authors referred to as the integration of an objective evaluation of one’s behavior with a social response. The data revealed an interesting pattern of attachment avoidance and anxiety being associated with opposite activation patterns in social-emotional information-processing areas (including the amygdala, caudate, anterior cingulate cortex, and anterior insula) when adolescents were exposed to incongruent social feedback. The authors discussed the findings in terms of attachment theory, particularly in relation to social adaptation through the engagement in, and resolution of, social conflict (Vrtička et al., 2014). Although these findings represent valuable preliminary evidence that attachment insecurity, and especially attachment avoidance, could (differentially) influence activity in brain areas sustaining social-emotional processing during adolescence, they do not provide any specific information on the underlying IWMs of self and others. This is due to the fact that attachment styles are conceptualized as prototypic strategies to regulate felt security (or the absence thereof) in social relationships, rather than representing general expectations about the worthiness of the self and the availability of others as the building blocks of self- and other-models (Bartholomew & Horowitz, 1991; Griffin & Bartholomew, 1994).

In contrast to the paucity of research in adolescents, a steadily growing number of brain-imaging studies investigating social-emotional processing as a function of attachment and, in most cases attachment style, is available in adults (DeWall et al., 2012; Donges et al., 2012; Lemche et al., 2006; Redlich et al., 2015; Strathearn, Fonagy, Amico, & Montague, 2009; Vrtička, Andersson, Grandjean, Sander, & Vuilleumier, 2008, Vrtička, Bondolfi, Sander, & Vuilleumier, 2012, Vrtička et al., 2014). The results of these studies are overall consistent with the attachment theory (Mikulincer & Shaver, 2007) in suggesting that de-activating strategies linked to attachment avoidance map to decreased brain activity, but hyper-activating strategies associated with attachment anxiety map to increased brain activity during attachment-related social-emotional information processing. Moreover, emerging evidence suggests that attachment avoidance may be particularly characterized by decreased reward-related activity to positive social stimuli in areas comprising the ventral tegmental area, ventral striatum, and medial orbitofrontal cortex (Strathearn et al., 2009; Vrtička et al., 2008), while attachment anxiety may be associated with increased threat- or rejection sensitivity-related activity to negative social stimuli in regions including the anterior cingulate cortex, insula, and amygdala (DeWall et al., 2012; Vrtička et al., 2008). Such notions based on neuroimaging results are corroborated by independent behavioral data (Rognoni, Galati, Costa, & Crini, 2008; Vrtička, Sander, & Vuilleumier, 2012). Other fMRI studies have investigated the effect of using implicitly or explicitly provided attachment security primes (i.e., words or images) on brain activity by itself (Canterberry & Gillath, 2013), in combination with social and linguistic threat (Norman, Lawrence, Iles, Benattayallah, & Karl, 2015), or during exposure to physical pain (Eisenberger et al., 2011). Finally, brain activation patterns as a function of attachment style have been assessed during different emotion-regulation paradigms (Gillath, Bunge, Shaver, Wendelken, & Mikulincer, 2005; Vrtička, Bondolfi, et al. 2012). Taken together, the available literature on associations between attachment style and brain activity in response to attachment-related social-emotional information in adults is beginning to reveal a coherent impression of the potentially maladaptive effects of attachment insecurity, as well as probable underlying neural patterns, particularly in the case of avoidance and positive social emotion processing (Vrtička & Vuilleumier, 2012). However, as is the case in adolescents, data specifically assessing self- and other-models as integral parts of IWMs is still crucially lacking (see above).

### Current investigation

To specifically assess the brain basis of self- and other-representation as a function of IWMs in adolescents, we set up the present fMRI paradigm based on an adapted version of the well-established trait-adjective evaluation task (TAET). During the TAET, participants are usually asked to indicate whether (i.e., yes or no) positive and negative trait words or phrases correspond to themselves, a familiar or a less familiar other, or to indicate to whom trait words or phrases correspond more (i.e., me vs. the other). Recent meta-analyses of fMRI studies in adults employing such TAET and closely related tasks confirm that involved neural computations consistently activate brain areas involved in social-emotional processing, such as the cortical midline structures, anterior cingulate cortex, and anterior insula (Murray, Debbané, Fox, Bzdok, & Eickhoff, 2015; van der Meer, Costafreda, Aleman, & David, 2010). These meta-analytic findings are complemented by other results from comparable tasks showing that a number of additional brain areas are differentially involved in positive and negative self- versus other-representation in adults, including the ventral striatum, amygdala, insula, precuneus, medial ventral, and dorsolateral prefrontal gyri, parahippocampus, supplementary motor area, and occipital cortex (Bruehl, Rufer, Kaffenberger, Baur, & Herwig, 2014; Cabanis et al., 2013; Pauly, Finkelmeyer, Schneider, & Habel, 2013; Yoshimura et al., 2009). Furthermore, neuroimaging data using the TAET is already available from children and adolescents and, despite exhibiting a mixed pattern across regions, suggests stronger recruitment of medial prefrontal cortex and temporal and parietal cortical regions during self-reflection, as well as ventral striatum during reflected social self-evaluation (Jankowski, Moore, Merchant, Kahn, & Pfeifer, 2014; Pfeifer, Lieberman, & Dapretto, 2007, Pfeifer et al. 2009). Overall, these fMRI findings regarding the usually applied TAETs and similar tasks in adults, adolescents, and children indicate that it is well suited for the purpose of the present investigation. It is important to note, however, that we used an adapted version of the TAET for the study at hand. One difference to most previous investigations was the choice of the other to be a close (same-sex) friend, rather than a familiar or less familiar/unknown person. This choice was motivated by the attachment context of the present study. Another difference was the adolescents’ tasks, which consisted of evaluating how well an adjective corresponds to the self or the close other on a scale from 1 to 4, rather than responding to the question to whom the adjective corresponds better, or whether the adjective corresponds to the self or the other at all.

In addition to employing a direct self-other trait evaluation task (i.e., an adapted version of the TAET), we calculated scores specifically reflecting participants’ attachment-derived self- and other-models by administering the Relationship Questionnaire (RQ; see Methods). Rather than using a measure assessing attachment styles pertaining to prototypic strategies aimed at regulating felt security (or the absence thereof) in social relationships, the derived self- and other-models directly represent a person’s view of the self and others in terms of expectations about the worthiness of the self and the availability of others (Bartholomew & Horowitz, 1991; Griffin & Bartholomew, 1994).

### Hypotheses

According to the available evidence, we predicted that attachment-derived representations of self and others would significantly modulate activity in the above-described social-emotional brain areas underlying the evaluation of positive and negative adjectives during our version of the TAET in adolescents.

The attachment-derived self-model reflects the positivity of a person’s self-concept, which (inversely) maps to the attachment anxiety dimension (Bartholomew & Horowitz, 1991; Griffin & Bartholomew, 1994; Mikulincer & Shaver, 2007). Consequently, we anticipated that adolescents with lower scores on the self-model would show increased activity in social-emotional brain areas during exposure to negative evaluations of the self, because such negative self-evaluations sustain negative self-representations.

In turn, the attachment-derived other-model reflects a person’s expectations about the availability of others, which (inversely) maps to the attachment avoidance dimension (Bartholomew & Horowitz, 1991; Griffin & Bartholomew, 1994; Mikulincer & Shaver, 2007). Accordingly, we anticipated that adolescents scoring low on the other-model would show decreased neural social-emotional processing of positive other-representations.

Finally, we were also interested in investigating whether there were any age-related effects within our sample of adolescents aged 12–18 years, as we have previously found an influence of age on brain activity in another sample of adolescents during a different fMRI task (Vrtička et al., 2014).

## Methods

### Participants

Healthy adolescents (n = 44; 23 female; age = 16 ± 1.86 years; range = 12.01–18.84) were included in the final data analysis of the present study. Participants were native French-speaking adolescents recruited from secondary schools in Geneva, Switzerland, by advertisements. Written informed consent was obtained from participants and their parents under protocols approved by the Institutional Review Board of the Department of Psychiatry of the University of Medicine, Geneva. Adolescents were remunerated for their participation.

A total number of n = 54 adolescent participants were recruited for the current investigation as part of a study on social cognition and adolescent brain development. Participants were recruited to written advertisements circulated in schools and public areas (community areas for youths, community sport centers, etc.). Before being considered eligible for the study at hand, potential participants were separately evaluated for fMRI exclusion criteria, which resulted in the n = 54 healthy adolescents admitted to this investigation. During the actual experiment, n = 3 individuals were excluded because they did not complete the paradigm, n = 1 had an incidental structural brain finding, n = 1 showed signs of substance abuse before entering the scanner, and n = 5 data sets showed excessive movement (values greater than 3 mm in translation or 3° in rotation) as detected during fMRI data pre-processing. The latter n = 10 participants were consequently removed, leaving a final number of n = 44 participants for data analysis. One participant had missing attachment questionnaire scores (see below), so that correlation analyses were performed in n = 43 participants.

### Questionnaires

All questionnaires were administered in French using validated French questionnaire versions. To derive participants’ internal (attachment) working models (IWMs) of self and others, we administered the RQ (Bartholomew & Horowitz, 1991; translated and validated by Guedeney, 2005). Because individual differences may be better explained by dimensional rather than categorical models (Fossati et al., 2003), we used continuous ratings of the four attachment descriptions of the RQ on a 7-point scale (1 = “this is not at all like me” to 7 = “this is absolutely like me”) to calculate two-dimensional values representing IWMs of self and others. According to Griffin and Bartholomew (1994), the self-model was computed as follows: rating scores of patterns characterized by a positive view of the self (i.e., secure and dismissing) minus rating scores of patterns characterized by a negative view of the self (i.e., fearful and preoccupied). Lower scores indicate consideration of the self as more negative and thus undeserving of help or love from close others. The other-model also consisted of a difference score and was calculated as follows: rating scores for patterns characterized by a positive view of others (i.e., secure and preoccupied), minus rating scores for patterns characterized by a negative view of others (i.e., fearful and dismissing). Lower scores designate a representation of significant others as being unhelpful and unreliable.

Participants also completed the Youth Self Report (YSR; Achenbach, 1991) or, for those > 18 years old, the Adult Self Report (ASR; Achenbach & Rescorla, 2003), to control for internalizing and externalizing problems. In addition, we obtained scores for schizotypy from the Schizotypal Personality Questionnaire (SPQ; total score; Badoud, Chanal, van der Linden, Eliez, & Debbané, 2011) and for borderline personality disorder from the Borderline Personality Inventory (BPI; total score; Chabrol et al., 2004) – these scores were used to control for potentially confounding personality variables (see Debbané et al., 2014).

Finally, to obtain an independent test of validity, we administered the Relationship Scales Questionnaire (RSQ) (Griffin & Bartholomew, 1994; Guedeney, Fermanian, & Bifulco, 2010), and analyzed it as reported previously (Vrtička, Bondolfi, et al. 2012, Vrtička, Sander, et al. 2012, Vrtička et al., 2014) by deriving continuous scores for each person on the two dimensions representing attachment avoidance and anxiety.

Participants completed all questionnaires in the week preceding their visit to the laboratory, together with other measures of personality.

### Stimuli

For our version of the TAET, we used adjectives taken from Anderson’s list of personality-trait words (Anderson, 1968). Selection was firstly based on the likeableness ratings (as reported by Anderson): the 169 most positive and 169 most negative adjectives were extracted. These adjectives were translated into French by two independent translators and non-concordant translations were solved by consensus. Adjectives that were translated with an identical word were discarded, and any adjective that was translated using paraphrases was also dismissed. Finally, using the most meaningful traits (Anderson, 1968), 55 positive-valence (e.g., sincere, polite, confident) and 55 negative-valence (e.g., hypocrite, cruel, clumsy) trait adjectives were selected. As the TAET comprised three experimental conditions (self/close other/syllable counting), the 55 adjectives per valence were then distributed to three separate lists, each comprising 25 adjectives (corresponding to five blocks of five adjectives). In so doing, ten adjectives were systematically repeated across the three lists, while the remaining 15 adjectives were different for each list. The three lists were randomized across participants. All adjectives and their distribution into the six lists (three per valence) can be found in the Supplemental Material.

### Experimental task

**Fig. 1:**
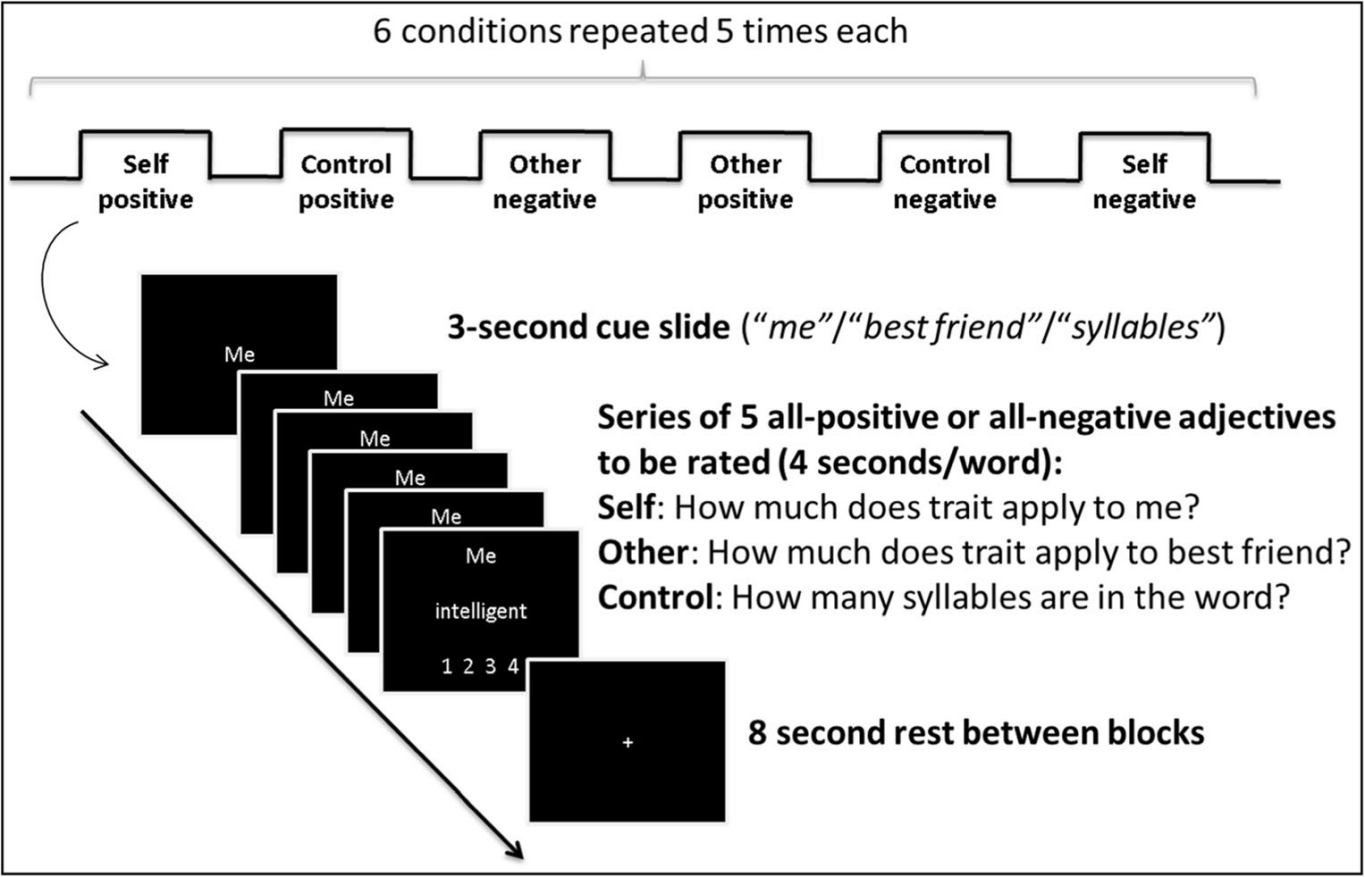
Illustration of the experimental paradigm. Participants had to attribute positive or negative adjectives to either themselves or a close other (best same-sex fried), or to count the syllables in the adjectives (six experimental conditions in total). We used a block design with each block comprising five adjectives shown for 4 s each. Every block was preceded by a cue slide shown for 3 s, announcing the task (self- or other-focus, or syllable counting), and followed by an 8-s rest period (fixation cross). Participants completed five blocks for each of the six experimental conditions, and block order was pseudo-randomized within and across participants

Stimuli were presented using E-Prime 2.0 software (Psychology Software Tools, Pittsburgh, PA, USA). During the block-designed fMRI paradigm, participants were asked to rate adjectives taken from the Anderson database (Anderson, 1968) referring to themselves (self condition) or their same-sex best friend (close-other condition) on a scale from 1 = “not at all” to 4 = “completely.” The control condition consisted of counting the syllables in each word (control condition) from 1 = “one syllable” to 4 = “four or more syllables.” At the beginning of each block, an instruction screen with the cue “me,” “best friend,” or “syllable” was shown for 3 s. Each block (with the cue remaining at the top of the screen) comprised five adjective ratings of 4 s each, with an 8-s resting period between blocks (see Fig. 1). The experiment consisted of one run comprising 30 blocks, ten per condition (self, close other, and control; five using positive and five using negative traits). The order of blocks was pseudo-randomized within and across participants. Total task time was approximately 16 min.

### Data acquisition and imaging

Participants were scanned at the Brain and Behaviour Laboratory (BBL) at the University of Geneva, using a 3-Tesla Trio MRI system (Siemens, Erlangen, Germany). Stimuli were presented on a screen at the back of the MRI tube, reflected in a mirror placed on the 12-channel head coil positioned above the participant’s head. A vacuum cushion was placed under the participant’s head to prevent excessive head movement. High-resolution structural T1-weighted images were obtained in one volume of 192 slices (TR = 2,500 ms, TE = 30 ms, slice thickness = 1.1 mm, flip angle = 8°, field of view (FOV) = 220 mm). Blood oxygenation level-dependent (BOLD) functional echo planar images (EPIs) consisted of 395 volumes, each comprising 38 slices (TR = 2,400 ms, TE = 30 ms, slice thickness = 3.2 mm, flip angle = 85°, FOV = 235 mm), obtained in a descending order (from top to bottom) parallel to the AC-PC line.

### Imaging analyses

Functional images were analyzed using SPM8 (Department of Neuroscience, Wellcome Trust Center for Neuroimaging, London, UK; http://www.fil.ion.ucl.ac.uk/spm/software/spm8/), running on Matlab (MathWorks, Inc., Natick, MA, USA). Images were visually inspected for potential signal loss due to magnetic field inhomogeneity. To correct for head motion, images were then realigned using a least squares approach and a six-parameter rigid-body registration. We subsequently performed slice-timing using the middle slice as a reference to correct for acquisition time differences, co-registration to individual structural images using a rigid body transformation in three dimensions, and normalization to the MNI (Montreal National Institute) space using a standard T1-weighted average scan provided by SPM8 (ICBM152; dimensions: 91 × 109 × 91; voxel size: 2 mm^3^; pre-smoothed to 8 mm). Finally, spatial smoothing with an 8-mm full width, half-maximum isotropic Gaussian kernel was performed, and a high pass filter was applied (cutoff 400 s – due to the pseudo-randomized order of conditions, the maximum time between two identical conditions (i.e., task-frequency) was ca. 350 s so that the cutoff was extended beyond the standard value of 128 s).

We modeled the following six conditions of interest at the single subject level as 20-s boxcar functions starting from the first word of five in each block, and convolved them with the standard hemodynamic response: self positive, close other positive, self negative, close other negative, control positive, and control negative. To capture variance due to movement, realignment parameters were incorporated as six additional regressors of no interest.

### Statistical analyses

#### Behavioral data

Behavioral data, consisting of adjective ratings and reaction times, were analyzed using SPSS (www.ibm.com/software/analytics/spss/, Version 22). First, two repeated measures analyses of variance (ANOVAs) with the factors focus (self vs. close other – in the case of reaction times also including syllable counting) and valence (positive vs. negative) were computed across the entire sample of participants. In a second step, and according to our hypotheses, we calculated five multiple regression analyses. The primary dependent variables were the behavioral/reaction time values representing the self versus close other difference scores, and this difference was subsequently decomposed into the following contrasts: (i) self positive versus close other positive, (ii) self negative versus close other negative, (iii) self positive versus self negative, and (iv) close other positive versus close other negative. Covariates of interest were the attachment-derived self- and other-models extracted from the RQ, and we controlled for age, sex, and internalization/externalization, borderline, and schizotypy scores. All continuous covariates were centered.

#### fMRI data

Functional MRI analyses were performed with SPM8 running under Matlab.

#### Task validation

For the purpose of task validation, comparisons reflecting contrasts between adjective attribution versus syllable counting (and vice versa) were computed first in terms of simple one-tailed t-tests with a combined statistical threshold of p < .001 uncorrected at the peak and p < .05 FWE-corrected at the cluster level (FWE-correction was determined by SPM).

#### Self- versus other-processing

According to behavioral data analysis, a repeated measures ANOVA with the factors focus (self vs. close other) and valence (positive vs. negative) was subsequently computed across the entire sample of participants (n = 44) by using a flexible factorial design (also comprising the factor subject). The only significant effect observed, namely the main effect of focus (i.e., self vs. close other; see Results below) was then further decomposed by one-tailed t-tests always contrasting two out of the four conditions (i.e., self positive vs. close-other positive and self negative vs. close-other negative). Again, a combined statistical threshold of p < .001 uncorrected at the peak and p < .05 FWE-corrected at the cluster level was applied (FWE-correction was determined by SPM). Significant activations derived from the main effect of focus were then compared to activations observed for the main effect of task adjective attribution > syllable counting to check whether they represented overlapping or distinct brain areas using the XjView SPM toolbox (http://www.alivelearn.net/xjview).

#### Effects of the self-and other-models on brain activity

According to our hypotheses, we then derived five whole-brain multiple regression analyses to test for specific effects of the attachment-derived self- and other-models on the main effect of focus and the four specific focus × valence interaction contrasts as its decomposition (i.e.: (i) self positive vs. close other positive, (ii) self negative vs. close other negative, (iii) self positive vs. self negative, and (iv) close other positive vs. close other negative). The whole-brain multiple regression analyses always comprised one given contrast (i.e., self vs. close other), were complemented with covariates of interest (i.e., the attachment-derived self- and other-models extracted from the RQ), and we controlled for age, sex, internalization/externalization, borderline, and schizotypy scores. Potential effects of covariates on brain activity were always assessed in both (i.e., positive and negative) directions. Once more, a combined statistical threshold of p < .001 uncorrected at the peak and p < .05 FWE-corrected at the cluster level was applied (FWE-correction was determined by SPM). Because we previously observed age-related differences in neural processing in adolescents (Vrtička et al., 2014), we also checked for potential age effects within the contrast self vs. close other (independent two-sample t-test also controlling for sex) and within the above described five multiple regression analyses. Significant activations derived from the whole-brain multiple regression analyses were then again compared to activations observed for the main effect of task adjective attribution > syllable counting to check whether they represented overlapping or distinct brain areas using the XjView SPM toolbox (http://www.alivelearn.net/xjview). Finally, because whole-brain correlations were always derived based on a contrast (i.e., self vs. close other), we were interested in finding out about the exact underlying conditions driving the observed effect at the contrast level. To this end, we extracted and averaged raw activation (betas) from all activation clusters and ran separate regressions in SPSS for each of the four experimental conditions.

## Results

### Demographic and behavioral data

Raw questionnaire scores (mean, range, and standard deviation) are summarized in Table 1. All scores were checked for outliers (applying the outlier labelling rule with q = 2.2 (Hoaglin & Iglewicz, 1987; Hoaglin, Iglewicz, & Tukey, 1986)), but there were none detected. There were no sex differences in any measures (p > .05).

**Table 1:** Raw questionnaire scores (n = 44, except for the RQ and RSQ where n = 43)

Measure (questionnaire)	Mean	Range	Standard deviation
Externalization (YSR/ASR)	54.18	30 to 67	9.22
Internalization (YSR/ASR)	50.32	30 to 69	9.74
Schizotypy (SPQ)	19.43	0 to 56	13.04
Borderline Personality (BPI)	99.91	51 to 188	34.39
Self-Model (RQ)	2.19	−9 to 8	4.19
Other-Model (RQ)	2.47	−4 to 7	2.73
Attachment Avoidance (RSQ)	18.21	11 to 34	4.52
Attachment Anxiety (RSQ)	11.56	5 to 25	4.41

Values are in the expected and previously reported range

*Used questionnaires:* Internalization/externalization – Youth Self Report (YSR; Achenbach, 1991) or, for those >18 years old, Adult Self Report (ASR; Achenbach & Rescorla, 2003); schizotypy – Schizotypal Personality Questionnaire (SPQ; total score; Badoud et al., 2011); borderline personality disorder – Borderline Personality Inventory (BPI; total score; Chabrol et al., 2004); attachment-derived self- and other-models – relationship questionnaire (RQ; Bartholomew & Horowitz, 1991; Guedeney, 2005); attachment style –Relationship Scales Questionnaire (RSQ ; Griffin & Bartholomew, 1994; Guedeney et al., 2010)

Simple correlation analysis revealed that the self-model was positively related to age (r = .360, p = .018), but negatively related to the schizotypy (r = −.342, p = .025) and borderline personality (r = −.346, p = .023) scores. No such correlations were observed for the other-model. The independent validation analysis with RSQ scores reflecting the dimensions of attachment avoidance and anxiety revealed that, as expected (Griffin & Bartholomew, 1994), the self-model was negatively correlated with anxiety (r = −.433, p = .004) and the other-model was negatively correlated with avoidance (r = −.487, p = .001). All intercorrelations between questionnaire scores are provided in Table 2.

**Table 2:** Intercorrelations between all questionnaire scores (n = 43)

		SELF	OTHER	AX	AV	EXT	INT	SPQ	BPI
SELF	Pearson r	1	−0.162	−.433**	−0.078	−0.213	−0.164	−.342*	−.346*
	p-Value		0.299	0.004	0.621	0.171	0.294	0.025	0.023
OTHER	Pearson r		1	−0.032	−.487**	0.04	−0.124	−0.058	−0.038
	p-Value			0.839	0.001	0.797	0.427	0.712	0.809
AX	Pearson r			1	0.122	.468**	.361*	.527***	.593***
	p-Value				0.436	0.002	0.017	<.001	<.001
AV	Pearson r				1	0.145	0.247	.327*	.324*
	p-Value					0.354	0.11	0.032	0.034
EXT	Pearson r					1	.549***	.554***	.722***
	p-Value						<.001	<.001	<.001
INT	Pearson r						1	.683***	.543***
	p-Value							<.001	<.001
SPQ	Pearson r							1	.741***
	p-Value								<.001
BPI	Pearson r								1
	p-Value								

*SELF* attachment-derived self-model, *OTHER* attachment-derived other-model, *AX* attachment anxiety, *AV* attachment avoidance, *EXT* externalization, *INT* internalization, *SPQ* schizotypal personality questionnaire (total score), *BPI* borderline personality inventory (total score)

* p < .05, ** p < .01, *** p < .001 (simple correlations, two-tailed)

### Behavioral data

All behavioral values were checked for outliers (applying the outlier labelling rule with q = 2.2 – see above), but there were none detected. For adjective ratings, behavioral data analysis across all participants (n = 44) revealed a main effect of valence (positive > negative; F = 284.83, p < .001) as well as a focus × valence interaction (F = 5.98, p = .019). The interaction arose because participants showed a slight close other-positivity bias. In other words, participants more readily attributed negative adjectives to themselves but positive adjectives to the close other (best same-sex friend). Regarding reaction times, data analysis across all participants (n = 44) showed a main effect of valence (F = 13.97, p < .001) because participants responded more slowly to negative than positive items.

**Fig. 2:**
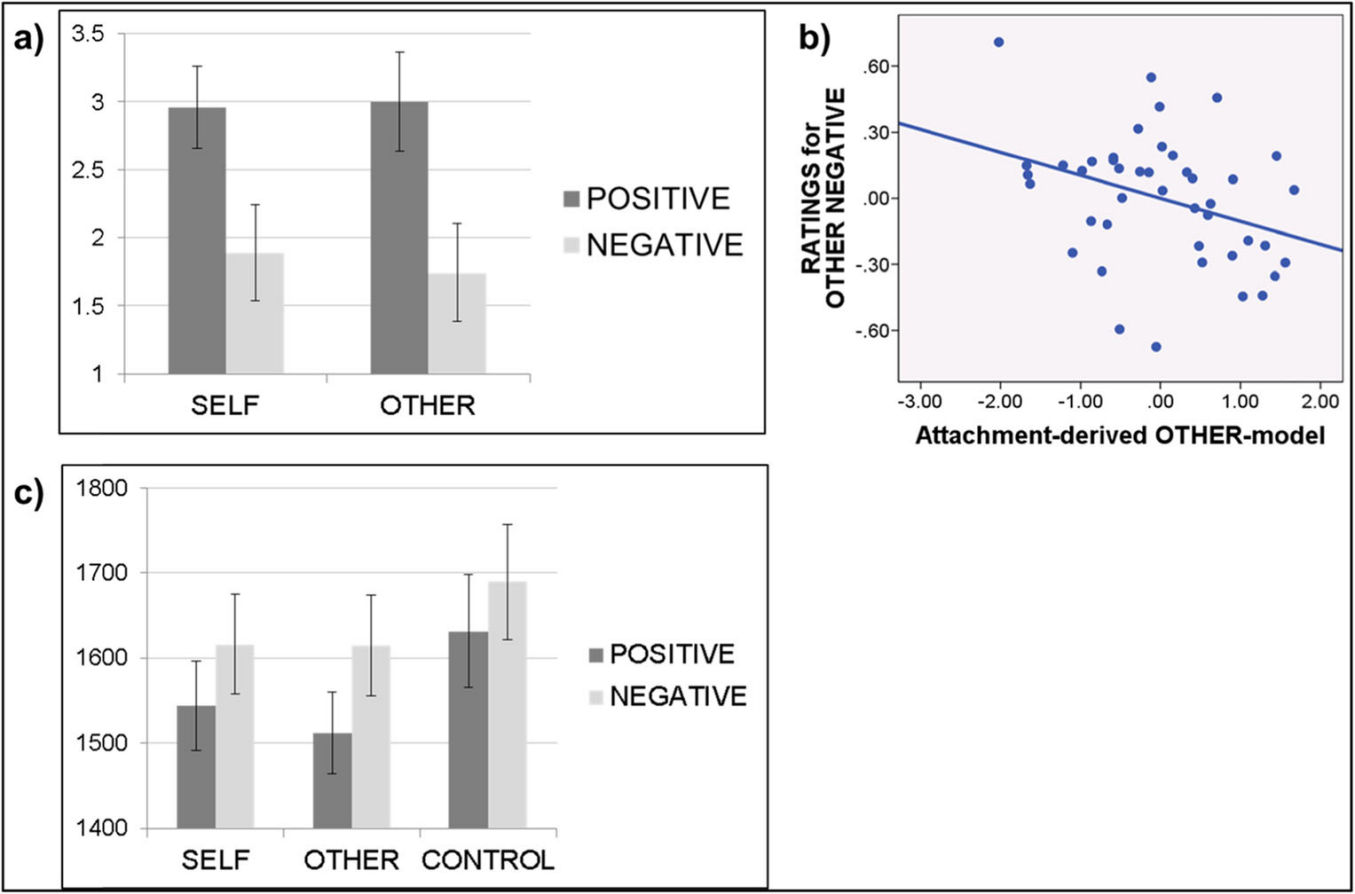
Behavioral data. (**a**) Rating scores during adjective attribution (on a scale from 1 to 4). (**b**) Negative correlation between rating scores for the negative other condition (y axis) and the attachment derived close other-model (x axis). (**c**) Reaction times (in milliseconds) during adjective attribution or syllable counting. Error bars indicate ± 1 standard deviation from the mean

When investigating the influence of the self- and other-models on adjective rating scores and reaction time by deriving multiple regression analyses (n = 43), a significant relation emerged only between the other-model and self negative versus close other negative difference scores (beta = .392, p = .012), which was driven by a simple negative association between the other-model and ratings for the close other negative condition (beta = −.293, p = .038): that is, the more positive the other-model, the less readily negative adjectives were attributed to the close other. Behavioral findings are summarized in Fig. 2. For the used regression models, tolerance values ranged from .296 (BPI total score) to .912 (attachment-derived other-model), with variance inflation factors ranging from 1.096 (attachment-derived other-model) to 3.377 (BPI total score).

### fMRI data

All random-effect models reported in this manuscript (see below) can be found on Neurovault: http://neurovault.org/collections/YCNBSMZQ/.

#### Task validation

**Fig. 3:**
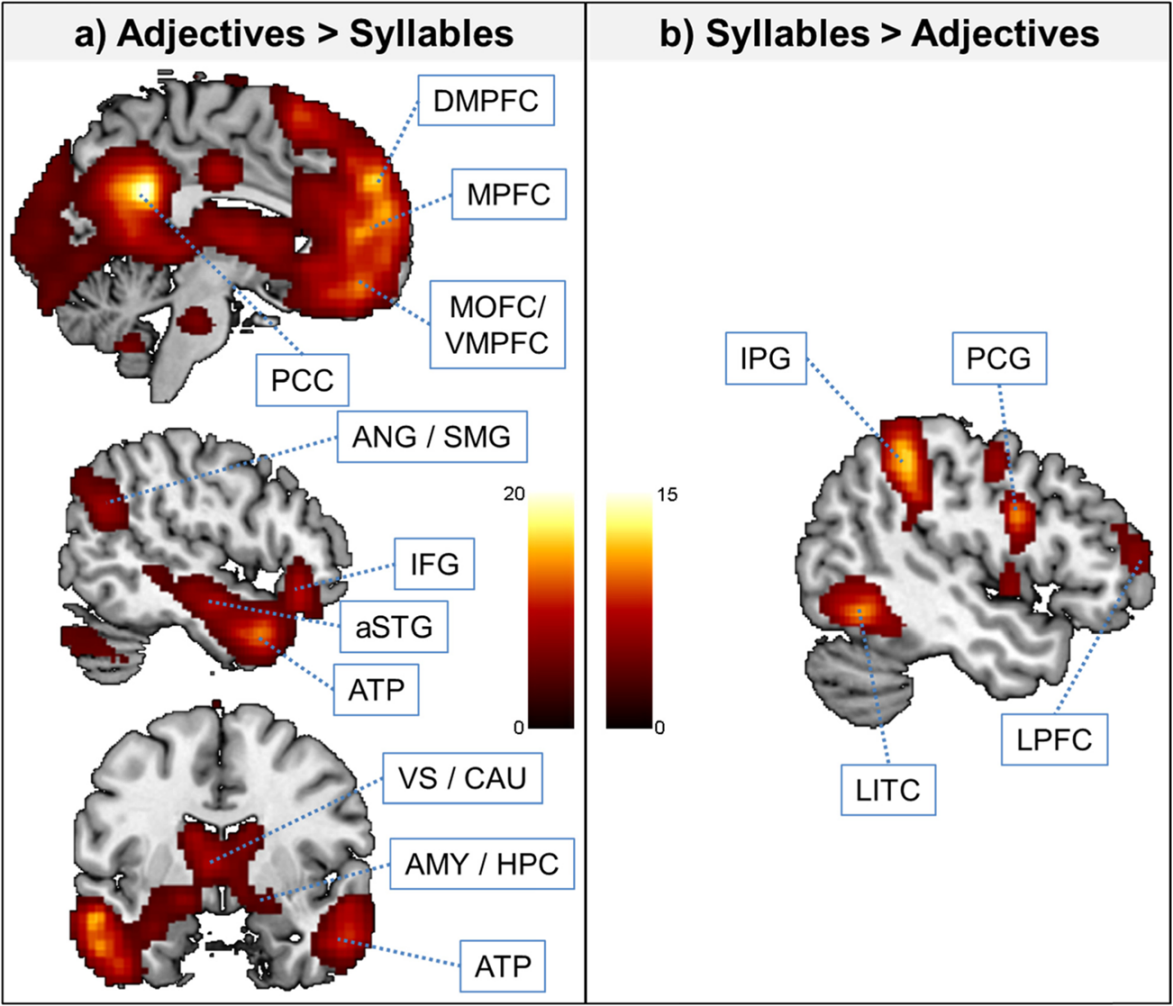
Main effects of fMRI analyses for adjective attribution versus syllable counting. Statistical threshold for activation maps is p < .001 uncorrected at the peak and p < .05 FWE-corrected at the cluster level. Activations are overlaid on a single-participant anatomical T1-weighted image. *MOFC* medial orbitofrontal cortex, *VMPFC* ventromedial prefrontal cortex, *ATP* anterior temporal pole, *aSTG* anterior superior temporal gyrus, *PCC* posterior cingulate cortex, *ANG/SMG* angular gyrus/supramarginal gyrus, *IFG* inferior frontal gyrus, *MOG/IOG* medial/inferior occipital gyrus, *VS/CAU* ventral striatum/caudate, *AMY/HPC* amygdala/hippocampus, *IPG* intraparietal gyrus, *LITC* lateral inferior temporal cortex, *LPFC* lateral prefrontal cortex

We first computed the main effects of task contrasts: adjective attribution > syllable counting, self positive > syllable counting positive, self negative > syllable counting negative, close other positive > syllable counting positive, and close other negative > syllable counting negative. All five contrasts revealed overlapping activity in a range of brain areas comprising the posterior cingulate cortex (PCC), bilateral prefrontal cortex (including ventral, medial/orbitofrontal as well as dorsomedial portions – MOFC/VMPFC, MPFC, and DMPFC), left dorsolateral PFC (DLPFC), bilateral temporal areas (anterior temporal pole, anterior superior temporal gyrus, posterior superior temporal sulcus, temporo-parietal junction, and supramarginal gyrus – ATP, aSTG, pSTS, TPJ, SMG), bilateral subcortical areas (amygdala, ventral striatum, caudate), pons, as well as cerebellum. We also computed all inverse contrasts reflecting the comparison between syllable counting > adjective attribution. Here, we observed overlapping activity in bilateral intraparietal sulcus (IPS), inferior temporal cortex (ITC), bilateral lateral prefrontal cortex (LPFC), as well as right precentral gyrus (PCG) and left cerebellum. Findings are summarized in Table 3 and Fig. 3 (illustrated is the contrast adjective attribution vs. syllable counting).

**Table 3:** Main effects fMRI contrast results for the comparisons between adjective attribution versus syllable counting, listing coordinates (x y z) and best estimates of anatomical peaks

p (FWE-cor. at the cluster level)	Voxel (k)	peak T	xyz	Region
Adjective Attribution > Syllable Counting				
<.001	72,306	20.45*	−4 −50 28	Posterior cingulate cortex
		17.82	−6 56 34	Dorsomedial prefrontal cortex
		17.69	−56 −6 −20	Left anterior superior temporal gyrus
		17.63	−42 16 −32	Left anterior temporal pole
		16.36	−46 22 −12	Left inferior frontal gyrus
		15.11	26 −80 −32	Right cerebellum
		14.92	−2 60 18	Medial prefrontal cortex
		13.76	−2 48 −16	Ventromedial prefrontal cortex
0.003	477	10.83*	8 −56 −42	Cerebellum
0.006	424	4.75	−40 −20 62	Left precentral gyrus
Syllable Counting > Adjective Attribution				
<.001	9,460	14.55*	−42 −42 44	Left intraparietal sulcus
<.001	1,357	12.99*	54 −58 −12	Right inferior temporal cortex
<.001	1,604	11.88*	−50 −60 −12	Left inferior temporal cortex
<.001	6,108	11.45*	50 −38 54	Right intraparietal sulcus
<.001	2,053	9.14*	44 6 28	Right precentral gyrus
<.001	1,066	7.32*	40 40 16	Right dorsolateral prefrontal cortex
0.038	271	6.18*	−28 −64 −28	Left cerebellum
<.001	722	5.66*	−48 48 10	Left dorsolateral prefrontal cortex
Self Positive > Syllables Positive				
<.001	61,451	18.91*	−4 −48 28	Posterior cingulate cortex
		16.43	−12 56 32	Dorsomedial prefrontal cortex
		15.95	−60 −16 −16	Left anterior superior temporal gyrus
		15.82	−42 20 −16	Left inferior frontal gyrus
		13.9	−4 60 20	Medial prefrontal cortex
		13.78	−46 16 −32	Left anterior temporal pole
		13.35	−2 50 −12	Ventromedial prefrontal cortex
		13.3	−48 −66 30	Left angular/supramarginal gyrus
		13.2	26 −78 −34	Right cerebellum
0.022	320	9.62*	8 −54 −42	Right cerebellum
<.001	1,228	8.48*	56 −62 30	Right angular/supramarginal gyrus
Syllables Positive > Self Positive				
<.001	4,257	12.03*	−40 −44 44	Left intraparietal sulcus
<.001	912	10.28*	54 −56 −12	Right inferior temporal cortex
<.001	4,666	9.25*	46 −40 52	Right intraparietal sulcus
<.001	1,116	9.23*	−50 −60 −12	Left inferior temporal cortex
<.001	975	8.7*	44 8 28	Right precentral gyrus
<.001	1,444	7.79*	−46 2 26	Left precentral gyrus
<.001	1,056	6.11*	40 36 16	Right dorsolateral prefrontal cortex
0.02	330	5.34	−40 52 24	Left dorsolateral prefrontal cortex
0.032	291	5.27	28 2 54	Right precentral gyrus
Self Negative > Syllables Negative				
<.001	49,962	16.91*	−6 −52 28	Posterior cingulate cortex
		14.7	−42 16 −32	Left anterior temporal pole
		13.96	−60 −16 −16	Left anterior superior temporal gyrus
		13.85	−12 46 42	Dorsomedial prefrontal cortex
		12.62	−44 22 −12	Left inferior frontal gyrus
		11.66	−2 60 16	Medial prefrontal cortex
		10.69	48 10 −34	Right anterior temporal pole
<.001	1,593	12.77*	26 −80 −32	Right cerebellum
0.001	569	9.58*	−28 −82 −32	Left cerebellum
0.019	327	8.74*	6 −56 −42	Right cerebellum
<.001	1,241	6.93*	48 −58 28	Right angular/supramarginal gyrus
Syllables Negative > Self Negative				
<.001	1,261	10.38*	−50 −60 −10	Left inferior temporal cortex
<.001	6,489	9.69*	−40 −46 44	Left intraparietal sulcus
<.001	5,022	9.35*	26 −62 46	Right intraparietal sulcus
<.001	1,015	9.1*	54 −56 −12	Right inferior temporal cortex
<.001	1,190	7.15*	−10 0 56	Dorsal anterior cingulate cortex/supplementary motor area
0.004	458	5.81*	48 42 22	Right dorsolateral prefrontal cortex
0.001	553	5.66*	48 8 28	Right precentral gyrus
Close Other Positive > Syllables Positive				
<.001	39,940	18.27*	−4 −48 28	Posterior cingulate cortex
		16.95	−6 56 34	Dorsomedial prefrontal cortex
		16.1	−60 −18 −16	Left anterior superior temporal gyrus
		13.55	−46 24 −12	Left inferior frontal gyrus
		12.78	0 50 −12	Medial prefrontal cortex
		12.54	−42 16 −30	Left anterior temporal pole
		12.2	60 −8 −16	Right anterior superior temporal gyrus
<.001	1,563	14.15*	28 −80 −32	Right cerebellum
<.001	2,081	12.48*	−48 −64 28	Left angular/supramarginal gyrus
<.001	2,157	10.73*	−28 −82 −32	Left cerebellum
<.001	926	9.43*	60 −62 26	Right angular/supramarginal gyrus
0.05	248	8.89*	8 −54 −42	Right cerebellum
0.021	313	7.63*	0 −14 38	Middle cingulum
0.002	510	5.21	−30 −14 72	Left precentral gyrus
Syllables Positive > Close Other Positive				
<.001	6,227	10.86*	−38 −44 44	Left intraparietal sulcus
<.001	1,509	9.45*	−50 −60 −12	Left inferior temporal cortex
<.001	1,562	9.31*	46 6 24	Right precentral gyrus
<.001	2,248	9.22*	−46 4 26	Left precentral gyrus
<.001	6,209	9.21*	54 −36 46	Right intraparietal sulcus
<.001	1,213	8.34*	56 −56 −12	Right inferior temporal cortex
<.001	1,051	6.72*	42 40 16	Right dorsolateral prefrontal cortex
<.001	902	6.47*	−6 2 56	Dorsal anterior cingulate cortex/supplementary motor area
<.001	1,046	6.44*	−42 46 6	Left dorsolateral prefrontal cortex
0.002	488	5.84*	28 2 56	Right precentral gyrus
Close Other Negative > Syllables Negative				
<.001	57,501	17.03*	−2 −50 28	Posterior cingulate cortex
		14.86	−6 54 34	Dorsomedial prefrontal cortex
		14.7	−58 −12 −18	Left anterior superior temporal gyrus
		13.02	−2 62 18	Medial prefrontal cortex
		12.23	−4 46 −16	Ventromedial prefrontal cortex
		12.06	−44 22 −14	Left inferior frontal gyrus
		11.95	32 −84 −32	Right cerebellum
		11.95	−38 18 −32	Left anterior temporal pole
0.006	469	6.67*	−2 −14 36	Middle cingulum
Syllables Negative > Close Other Negative				
<.001	3,504	8.9*	−42 −42 44	Left intraparietal sulcus
<.001	1,018	8.56*	−50 −60 −12	Left inferior temporal cortex
<.001	3,925	8.54*	50 −40 56	Right intraparietal sulcus
0.001	660	8.37*	54 −56 −10	Right inferior temporal cortex
<.001	749	7.47*	−46 2 26	Left precentral gyrus
0.005	482	5.87*	46 6 28	Right precentral gyrus

For large clusters, the main peak plus unique sub-peaks as provided by SPM8 are listed

Asterisks (*) indicate clusters where the peak T-value also survived a FWE-correction (p < .05) at the voxel level. The final voxel size after preprocessing was 2 mm^3^

#### Self- versus other-processing

**Fig. 4:**
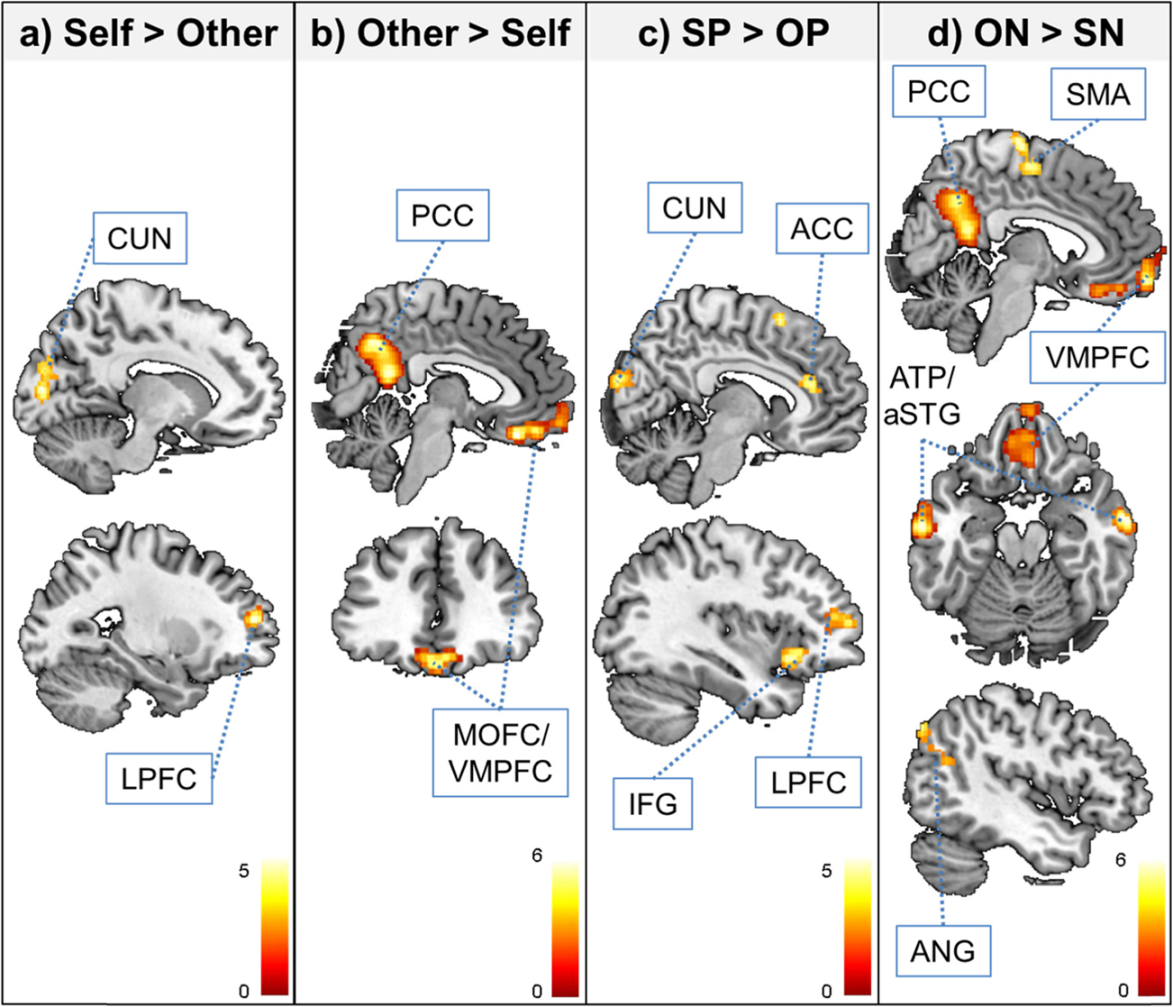
Main effects of fMRI analyses during adjective attribution. Statistical threshold for activation maps is p < .001 uncorrected at the peak and p < .05 FWE-corrected at the cluster level. Activations are overlaid on a single-participant anatomical T1-weighted image. *SP* self positive, *OP* close other positive, *SN* self negative, *ON* close other negative, *CUN* cuneus, *PCC* posterior cingulate cortex, *MOFC* medial orbitofrontal cortex, *VMPFC* ventromedial prefrontal cortex, *ACC* anterior cingulate cortex, *IFG* inferior frontal gyrus, *LPFC* lateral prefrontal cortex, *ATP* anterior temporal pole, *aSTG* anterior superior temporal gyrus, *ANG* angular gyrus

The repeated measures ANOVA (flexible factorial design also including the factor subject) only revealed a significant main effect of focus (i.e., self vs. close other). For the comparison self > close other, we observed increased activity in left lateral PFC (LPFC) and left cuneus. Further decomposition of this self > close other main effect of focus only revealed significant activation differences for the contrast self positive > close other positive, again in left LPFC and cuneus, and additionally in bilateral inferior frontal gyrus (IFG), anterior cingulate cortex (ACC), and DMPFC/pre-supplementary motor area (SMA). Conversely, for the comparison close other > self, we found increased activity in MOFC/VMPFC and PCC. Further decomposition of this close other > self main effect of focus only revealed significant activation differences for the contrast close other negative > self negative, again in MOFC/VMPFC and PCC, and additionally in bilateral ATP/aSTG, left angular gyrus (ANG), as well as SMA. All of the above-reported activations for the main effect of focus and its decompositions overlapped with activations observed for the main effect of task adjective attribution > syllable counting, except for the pre- SMA. Results are summarized in Table 4 and Fig. 4.

**Table 4:** fMRI contrast results for the comparisons during adjective attribution, listing coordinates (x y z) and best estimates of anatomical peaks

p (FWE-cor. at the cluster level)	Voxel (k)	peak T	xyz	Region
Self > Close Other				
0.022	326	5.35	−26 54 14	Left lateral prefrontal cortex
0.011	386	4.67	−12 −88 10	Left Cuneus
Close Other > Self				
<.001	1,324	6.78*	2 −50 18	Posterior cingulate cortex
<.001	746	6.22*	4 34 −22	Medial orbitofrontal/ventromedial prefrontal cortex
Self Positive > Close Other Positive				
0.006	416	5.36	−24 52 14	Left lateral prefrontal cortex
<.001	859	4.87	8 −92 22	Cuneus
0.002	530	4.84	−40 18 −8	Left inferior frontal gyrus
0.03	291	4.78	54 20 −16	Right inferior frontal gyrus
0.034	282	4.7	10 34 16	Anterior cingulate cortex
0.035	279	4.44	−12 22 58	Dorsomedial prefrontal cortex/pre-SMA
Close Other Negative > Self Negative				
<.001	1,017	6.4*	2 62 −12	Medial orbitofrontal/ventromedial prefrontal cortex
<.001	1,881	6.37*	2 −52 20	Posterior cingulate cortex
=.001	656	6.2*	−62 −10 −16	Left ATP/aSTG
0.005	454	5.54*	60 −4 −8	Right ATP/aSTG
0.018	337	4.84	−44 −78 42	Left angular gyrus
<.001	700	4.6	4 −18 72	Supplementary motor area

Only significant comparisons are reported

*SMA* supplementary motor area, *ATP* anterior temporal pole, *aSTG* anterior superior temporal gyrus. Asterisks (*) indicate clusters where the peak T-value also survived a FWE-correction (p < .05) at the voxel level. The final voxel size after preprocessing was 2 mm^3^

#### Effects of the self-model on brain activity

The subsequent whole-brain multiple-regression analyses tested for associations between brain activity and attachment-derived self- and other-models extracted from the RQ as covariates of interest. Significant effects were only observed for the main effect of focus contrast self versus close other in terms of a negative association with the attachment-derived self-model. A complete list of significant activations is provided in Table 5.

**Table 5:** Whole brain multiple regression fMRI analysis results, listing coordinates (x y z) and best estimates of anatomical peaks

p (FWE-cor. at the cluster level)	Voxel (k)	peak T	xyz	Region
Self versus Other × Self-Model Negative				
<.001	5,344	7.15*	16 −38 −32	Cerebellum/FFA/pons
0.004	434	5.31	22 −76 24	Right (pre)cuneus
0.067	223	5.21	−60 −4 −32	Left ATP
<.001	908	5.19	−28 −28 −22	Left hippocampus/amygdala
0.028	285	4.86	58 −2 −20	Right ATP/aSTG
0.061	230	4.47	−24 38 38	Left dorsolateral prefrontal cortex

*FFA* fusiform face area, *ATP* anterior temporal pole, *aSTG* anterior superior temporal gyrus. Asterisks (*) indicate clusters where the peak T-value also survived a FWE-correction (p < .05) at the voxel level. The final voxel size after preprocessing was 2 mm^3^

**Fig. 5:**
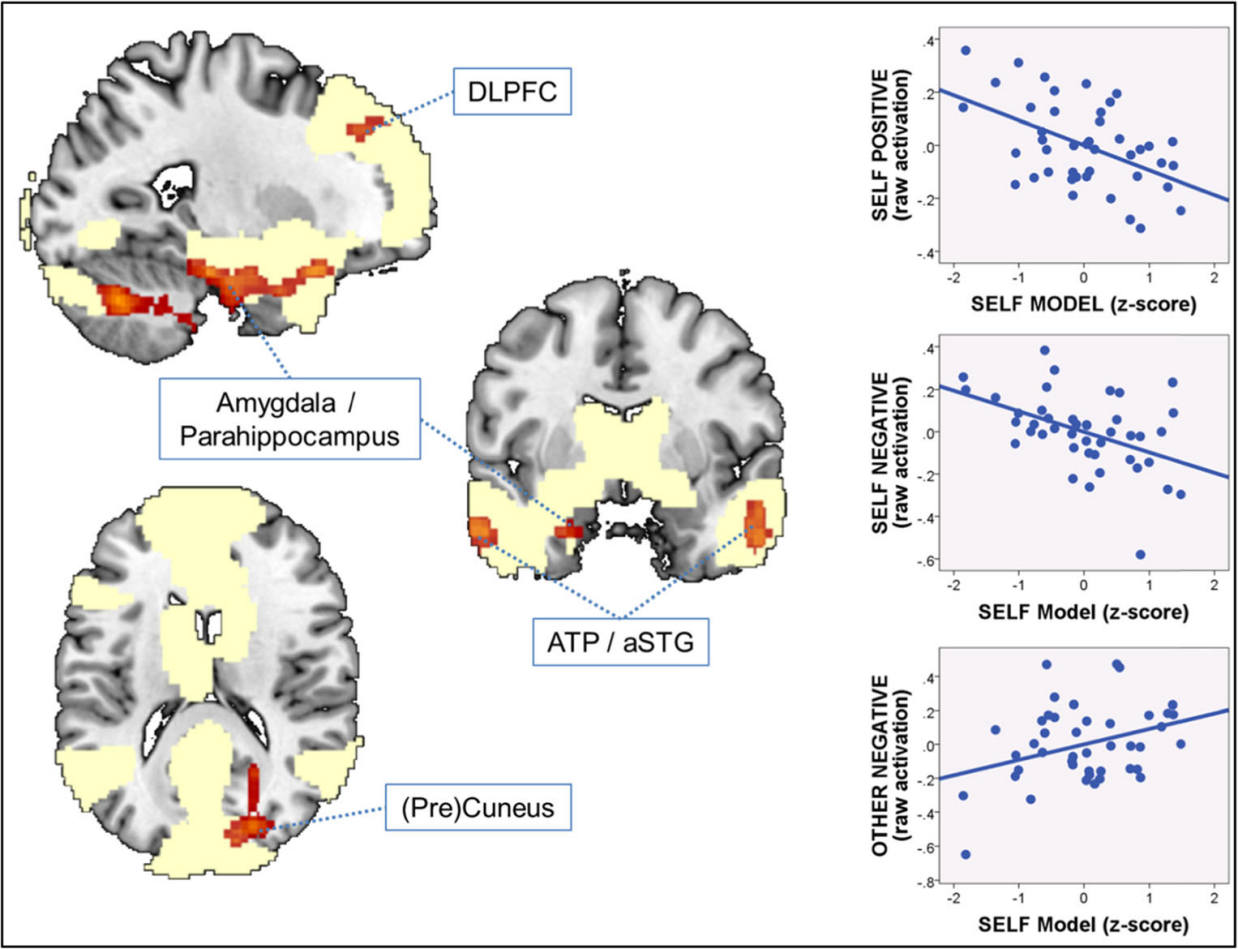
Whole brain multiple regression analysis depicting negative associations between the attachment-derived self-model and brain activity for the contrast “self versus close other” overlapping with adjective attribution areas. Statistical threshold for activation maps is p < .001 uncorrected at the peak and p < .05 FWE-corrected at the cluster level (except for left DLPFC where FWE p = .061, and left ATP where FWE p = .067). Activations are overlaid on a single-participant anatomical T1-weighted image and superimposed on the main effect of task contrast adjective attribution > syllable counting (light yellow). *DLPFC* dorsolateral prefrontal cortex, *ATP* anterior temporal pole, *aSTG* anterior superior temporal gyrus. The three plots on the right depict the decomposed relations between brain activity for the self positive (*top*), self negative (*middle*), and close other negative (*bottom*) condition on the y-axis, and the attachment-derived self-model (x-axis) extracted from the left amygdala/hippocampus cluster (please refer to Table 5 for statistical values) that overlap with activation clusters for the main effect of task adjective attribution > syllable counting

In analyzing the above-described activation pattern, we first concentrated on activation clusters that lay (partially) within the main effect of task “adjective attribution > syllable counting” and thus represent a differential recruitment (as a function of the attachment-derived self-model) of brain areas usually employed during self- and other-representation. Brain areas that were activated during adjective attribution across the entire sample of participants and in addition showed a negative association with the attachment-derived self-model were the left amygdala/hippocampus, right (pre)cuneus, left LPFC (FWE p = .061), as well as bilateral ATP/aSTG (left ATP FWE p = .067). We then decomposed these significant whole-brain regressions between the self-model and the contrast self versus close other into the four individual experimental conditions by means of post-hoc multiple regression analyses on extracted (and averaged) raw activation (beta) values. These analyses revealed that the negative relation between the self-model and brain activity for the contrast self versus close other was driven by two effects: (i) increased BOLD signal change during the two self conditions (self positive and self negative) with lower self-model scores, and (ii) decreased BOLD signal change during the close other negative condition with lower self-model scores. Findings are summarized in Table 6, and an illustration of these effects, derived from the right amygdala/parahippocampus, is shown in Fig. 5.

**Table 6:** Decomposition of whole-brain multiple regression fMRI analysis results with the attachment-derived self-model

Region	SP	SN	OP	ON
Decomposition of Correlations with Self-Model overlapping with Adjective Attribution > Syllable Counting Areas				
Left dorsolateral prefrontal cortex	β = −.428, p = .025	β = −.432, p = .026	n.s.	β = .393, p = .027
Left parahippocampus/amygdala	β = −.471, p = .003	β = −.455, p = .006	n.s.	β = .348, p = .043
Right ATP	β = −.527, p = .001	β = −.539, p = .001	n.s.	n.s.
Left ATP	β = −.598, p < .001	β = −.434, p = .013	n.s.	n.s.
Left (Pre)Cuneus	n.s.	n.s.	n.s.	n.s.
Decomposition of Correlations with Self-Model in Additional Areas				
Cerebellum/FFA	β = −.524, p = .001	β = −.439, p = .016	n.s.	β = .405, p = .018

*IFG* inferior frontal gyrus, *ATP* anterior temporal pole, *FFA* fusiform face area

**Fig. 6:**
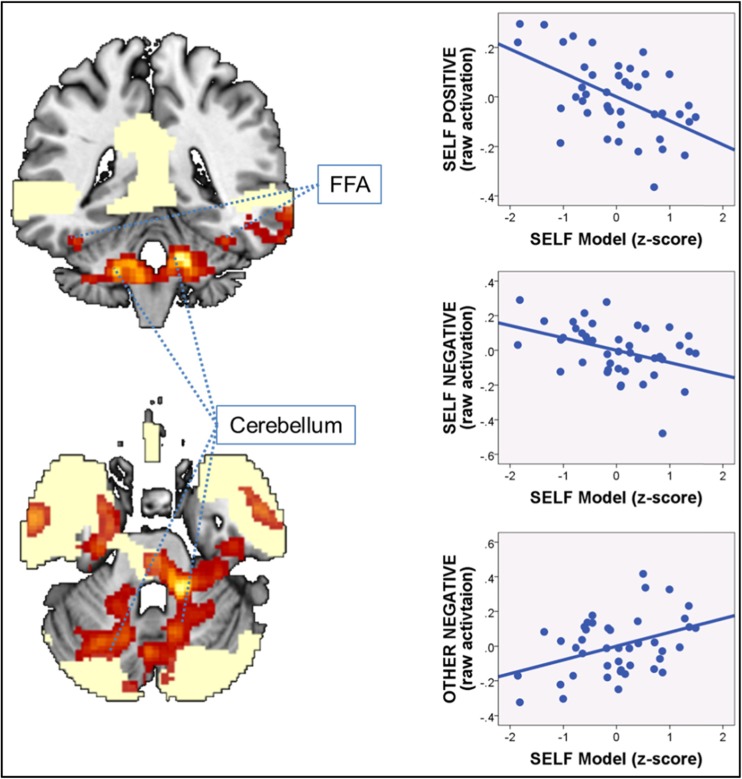
Whole brain multiple regression analysis depicting negative associations between the attachment-derived self-model and brain activity for the contrast “self versus close other” in additional areas. Statistical threshold for activation maps is p < .001 uncorrected at the peak and p < .05 FWE-corrected at the cluster level. Activations are overlaid on a single-participant anatomical T1-weighted image and superimposed on the main effect of task contrast adjective attribution > syllable counting (light yellow). *FFA* fusiform face area. The three plots on the right depict the decomposed relations between brain activity for the self positive (*top*), self negative (*middle*), and close other negative (*bottom*) condition on the y-axis, and the attachment-derived self-model (x-axis) extracted from the cerebellum/FFA cluster (please refer to Table 5 for statistical values) that does mostly not overlap with activation clusters for the main effect of task adjective attribution > syllable counting

We then also assessed whether there were any additional regions where the attachment-derived self-model influenced brain activity during adjective attribution, even though these areas were not generally more responsive during adjective attribution > syllable counting. This step revealed one extensive cluster spanning bilateral cerebellum, extending into bilateral fusiform face area (FFA) and pons. Although there was some overlap with the initial contrast adjective attribution > syllable counting (particularly in the pons), most of this extensive cluster did not show such overlap. Decomposition of the self versus other contrast used for the whole-brain multiple regression analysis into the four experimental conditions in this one extensive cluster revealed the same two underlying effects as already observed before: (i) increased BOLD signal change during the two self conditions (self positive and self negative) with lower self-model scores, and (ii) decreased BOLD signal change during the close other negative condition with lower self-model scores. Findings are summarized in Table 6, and an illustration is provided in Fig. 6.

When computing the above post-hoc regression analyses, we observed that in very few cases (at most one participant per experimental condition per region of interest, or 2.27% of data), raw activation (beta) values for specific experimental conditions were relatively low/high (identified as outliers when applying the outlier labeling rule with q= 2.2 – see above), while the beta values for the remaining experimental conditions were in the “normal” range. The affected low/high values were therefore not removed but winsorized to the respective low/high boundaries (the latter determined as the 25th or 75th percentiles ± 2.2 times the 25th–75th percentile range). Table 6 and the illustrations in Figs. 5 and 6 were derived from analyses including such winsorized values.

#### Effects of age on brain activity

Finally, we also investigated whether there were any age effects by assessing relations between brain activity and participant age within the contrast self versus close other and the five whole brain multiple-regression analyses as described above. However, these analyses did not reveal any significant effects.

## Discussion

This fMRI study aimed at investigating the neural basis of self- versus close other-representations reflecting IWMs of attachment in adolescents. Participants were asked to evaluate how well positive and negative adjectives corresponded to either themselves or a close other (best same-sex friend) while undergoing fMRI scanning. In subsequent analyses, brain activation was associated with participants’ attachment-derived self- and other-models. Results revealed significant relations between the self-model and brain activity in left amygdala/parahippocampus, bilateral ATP/aSTG, (pre)cuneus, left DLPFC, and cerebellum comparing self versus close other adjective evaluations. This activation pattern is discussed from an attachment theory perspective in the following sections, particularly reflecting upon adolescence as an important social-emotional developmental transition phase.

### General behavioral and fMRI task-related effects

To validate the use of our adjective evaluation versus syllable counting paradigm as a derivate of the TAET, we first assessed behavioral results and inspected main fMRI contrasts across the entire sample of 44 participants.

An interesting pattern emerged for the rating scores, in that there was a slight close other-positivity bias: rating scores were higher during the positive close other (vs. self) condition, but lower during the negative close other (vs. self) condition (valence × focus interaction). In other words, participants were more likely to attribute positive traits and less likely to attribute negative traits to their close other (vs. to themselves). This behavioral rating pattern suggests that participants generally maintained a positive close-other-representation, which was also reflected by a negative correlation between ratings during negative adjective attribution to the close other and other-model scores (i.e., the less readily participants attributed negative adjectives to their close other, the more positive was their other-model). In the psychology literature, there is strong evidence for a self-serving attributional and self-positivity bias, or put differently, the tendency to see the self as positive as such, as well as better than others (for reviews, see Taylor & Bown, 1988; Mezulis, Abramson, Hyde, & Hankin, 2004). Interestingly, however, the self-positivity bias has been shown to be somewhat attenuated when the other is a close friend, a pattern thought to emerge from the general inclination to appraise the self and one’s close associates as more positive (and less negative) than most other people. Furthermore, the self-serving attributional bias has been found to show an intriguing developmental pattern, being highest in children (ages 8–11 years), decline during early adolescence, remain lower during late adolescence and early to middle adulthood, and to increase again in late adulthood (ages 55 years and beyond). Possible reasons for a drop in self-serving attributional bias during early adolescence are discussed as including an increase in negative life events and the emergence of the cognitive ability to infer that negative events may be caused by internal, stable, and global causes such as a lack of ability (Taylor & Bown, 1988; Mezulis et al., 2004). Because in the present study the other was the participants’ best (same-sex) friend, participants may have had a particularly positive appraisal of him/her, entailing an abolishment (and even reversal) of the self-positivity bias. However, such pattern may also be more generally reflecting decreased self-serving attributional bias in adolescents. Our behavioral data therefore show that it may be beneficial to also include a close other condition in future investigations employing the TAET or related tasks because the appraisal of a close other may substantially differ from the appraisal of a familiar or unfamiliar/unknown other. An interesting future avenue of research may also be to investigate self- versus other-representation longitudinally to see whether only the self-serving attributional bias drops in adolescents, or whether there is also a change in the positivity of other-representations, again as a function of different degrees of other-closeness.

With regard to the fMRI results, our main effects across the entire sample of adolescents revealed that adjective attribution (vs. syllable counting) was associated with increased activity in an extended network of brain areas comprising cortical midline structures, bilateral temporal cortex, as well as bilateral subcortical/limbic regions. Furthermore, for the comparisons within adjective attribution conditions, we observed increased activity in the orbitofrontal/ventromedial prefrontal cortex and posterior cingulate cortex for the contrast close other > self (mirrored for the contrast close other negative > self negative), and in the cuneus, anterior cingulate cortex, inferior frontal gyrus as well as lateral prefrontal cortex for the contrast self positive > other positive. Activations within adjective attribution conditions overlapped to a large degree with activations for the main effect of task adjective attribution > syllable counting. These findings are in general agreement with previously published data on self- and/versus other-TAETs in both adolescents and adults, although the available data exhibit a mixed pattern across regions (see e.g., Debbané et al., 2014; Jankowski et al., 2014; Murray, Schaer, & Debbané, 2012; Pfeifer et al., 2007, 2009). The heterogeneous nature of so far reported effects of the TAET and similar tasks can partly be explained by the choice of different close, familiar or unfamiliar/unknown others, as well as different task instructions – e.g., the question whether an adjective corresponds more to the self or the other (i.e., response self or other), or whether an adjective corresponds to the self or the other at all (i.e., response yes or no). For example, evaluating which of two traits better describes the self versus the other is a task that may engage more relational reasoning than evaluating whether an adjective describes the self or the other at all. Furthermore, the self-relatedness and/or closeness of the other may also influence behavioral as well as brain responses. A recent meta-analytic review of 25 functional neuroimaging studies in adults (Murray et al., 2012) specifically investigated the difference in brain activity during TAETs and similar tasks as pertaining to the comparison of the contrasts self versus close other and self versus public other, as well as the contrasts close other versus control and public other versus control. Most importantly, an MPFC dorsal-ventral distinction was observed because brain activity for public other versus control was significantly more dorsal than brain activity for close other versus control. Due to the attachment context of the present investigation, the other was specifically chosen to be a close (same-sex) friend. In addition, our task involving a correspondence judgment on a Likert-scale from 1 to 4 deviated to some degree from the usually employed tasks. However, as described above, our findings fit well within the overall body of available TAET literature. Future studies directly comparing different tasks and manipulating the degree of self-relatedness of the other (or explicitly asking for a rating of self-other similarity) are nonetheless warranted and will help further clarifying the observed behavioral and brain patterns.

### Specific effects of the self-model on brain activity during the TAET

Our fMRI analyses regarding the relation between the attachment-derived self- and other-models and brain activity during our version of the TAET revealed significant effects only for the self-model within the contrast self versus close other. Further decomposition of this activation pattern revealed that the self-model was overall negatively related to BOLD signal change during the positive and negative self conditions, but positively related to BOLD signal change during the negative close other condition. To the best of our knowledge, these results represent the first available data – in adolescents or adults – showing a possible association between an attachment-derived IWM of self and brain activity. They therefore constitute an important addition to the extant attachment literature which mainly describes relations between brain activity and (anxious) attachment style.

#### Self-model and brain activity during adjective attribution to the self

On the one hand, our results suggest that adolescents who hold a negative attachment-derived view of themselves (i.e., low positivity of self-concept) show increased brain activity in the left amygdala/parahippocampus, bilateral ATP/aSTG, (pre)cuneus, and left DLPFC, while attributing positive and negative adjectives to themselves. Because activations in the above areas overlapped with activations for the main effect of task adjective attribution > syllable counting, this pattern suggests that a low positivity of self-concept is associated with differential recruitment of brain areas generally involved in adjective attribution when affected participants think about themselves.

Previous studies have described associations between amygdala activity and attachment anxiety during the processing of negatively valenced social images, social threat, or social punishment (Norman et al., 2015; Redlich et al., 2015; Vrtička et al., 2008, Vrtička, Bondolfi, et al. 2012). According to the general relevance detector account of human amygdala function (Pessoa & Adolphs, 2010; Sander, Grafman, & Zalla, 2003), these findings are thought to reflect increased salience of negatively valenced social information for anxiously attached individuals. Importantly, in the present study, we did not rely on scores reflecting attachment anxiety, but on a negative self-model in terms of IWMs (see above). Therefore, our new data add negative self-reflections to the relevant stimuli associated with increased amygdala activity in participants with a negative attachment-derived self-model. However, our data also revealed an association between amygdala activity during positive self-reflections and a negative attachment-derived self-model. According to the above definition of amygdala function as a relevance detector, it therefore seems that for adolescents with a more negative self-representation, self-evaluation in both positive and negative terms may constitute a highly salient process. Although not directly related to amygdala activation, it has previously been shown that attachment anxiety is also associated with increased neural activation (in left inferior, middle, and medial prefrontal areas, globus pallidus, claustrum, and right cerebellum) during the processing of positive social stimuli, in this specific case masked happy faces (Donges et al., 2012). Such an activation pattern was described as reflecting a motivation to achieve intimacy and approval in relationships while at the same time being mistrustful of others and their availability, a combination of factors leading to the involuntary dedication of more resources to the perception and evaluation of approach-related signals. Overall, our new data could indicate that the predisposition of anxiously attached individuals to automatically perceive positive social signals may also apply for more inward-oriented positive self-representations, particularly if the underlying attachment orientation is characterized by a negative self-model, and may manifest itself by increased amygdala activation reflecting heightened relevance attribution.

Similar to the above-described amygdala findings, we also observed a positive relation between the degree of attachment-derived self-model negativity and brain activity during the positive and negative self conditions in the anterior temporal pole (ATP), anterior superior temporal gyrus (aSTG), and the parahippocampus. Although these three areas serve distinct functions, they share one particular property, namely their involvement in embedding of emotional information into a social (attachment-related) context. On the one hand, recent accounts conceptualize the ATP as sustaining semantic aspects of emotionally tagged social knowledge. Such “semantic social knowledge” is thought to guide orbitofrontal-based decision processes through connections with the amygdala, independently of valence (Olson, McCoy, Klobusicky, & Ross, 2013). This recent account of ATP function is somewhat more general than results of earlier studies, which suggested that increased BOLD signal change in ATP was related to the processing of sadness (Gillath et al., 2005; Levesque et al., 2003), especially when participants with high attachment anxiety scores were asked to think about negative relationship scenarios (i.e., conflict, break-up, death of partner; Gillath et al., 2005). With regard to the present study, we may therefore hypothesize that, when confronted with representations of themselves, adolescents with a negative self-model could show an increased tendency for social contextualization of self-views, independent of their valence. On the other hand, Gillath et al. (2005) also described heightened attachment anxiety as sustaining increased hippocampal activity during negative relationship scenarios. The authors discussed such a pattern in the context of recalling negative attachment-related memories. Interestingly, the parahippocampus has been particularly implicated in the recall of context-related as opposed to item-related memory (Hannula, Libby, Yonelinas, & Ranganath, 2013; Wang, Yonelinas, & Ranganath, 2013). Our findings of increased parahippocampus activation as a function of a negative attachment-derived self-model partly mirror these findings, and may therefore reflect increased contextual attachment-related memory retrieval during the processing of negative self-attributions. However, our data also reveal increased parahippocampus activation during attribution of positive adjectives to the self as a function of increasing self-model negativity. It could therefore be that adolescents with a negative self-view more generally evaluate themselves in an attachment-related (social) context, independent of the valence of self-reflections. Furthermore, we also observed heightened right aSTG activation during positive and negative adjective self-attribution the more pronounced the adolescents’ self-model negativity was. The aSTG is thought to play an important role in social emotional processing (Vrtička, Sander, & Vuilleumier, 2013; Wicker, Perrett, Baron-Cohen, & Decety, 2003) more generally, and in the representation of abstract social concepts/values and moral sentiments (Zahn et al., 2007) as well as moral cognition (Moll, Zahn, de Oliveira-Souza, Krueger, & Grafman, 2005) more specifically. Although we are not aware of any specific findings linking aSTG activity to attachment insecurity during social emotional processing, our aSTG findings accord with the pattern observed in the ATP and parahippocampus, and may therefore also indicate an increased tendency for individuals with a negative attachment-derived self-model to evaluate themselves more strongly in a social context based on social values and moral sentiments.

In addition, the same general activation pattern reflecting increased brain activity during self-representation was present in the (pre)cuneus – although for this particular area, no experimental condition appears to have been specifically driving this overall effect. In the neuroscientific attachment literature, (pre)cuneus activation is not prominently reported. However, one study found increased (pre)cuneus activation investigating grief through the exposure of bereaved women to pictures of their deceased loved one (Gündel, O’Connor, Littrell, Fort, & Lane, 2003). Such activation was linked to heightened memory-related imagery possibly comprising face processing in a social (negative) context, a process that could also have been increased during the self-representation conditions in adolescents with a negative self-model in our study.

Finally, the same pattern of increased activity during the positive and negative self conditions as a function of self-model negativity was present in the left dorsolateral prefrontal cortex (DLPFC). Generally speaking, the DLPFC is though to be prominently involved in cognitive control processes like the coordination of thoughts and actions in accordance with overarching internally represented goals. These processes are often implicated in emotional control, particularly the down-regulation of negative emotion (Ahmed, Bittencourt-Hewitt, & Sebastian, 2015; Davidson, Putnam, & Larson, 2000). Preliminary evidence suggests that attachment insecurity is linked to altered prefrontal cortical activity, including in the DLPFC, during cognitive control/emotion regulation (Buchheim et al., 2006; Coan, Schaefer, & Davidson, 2006; Gillath et al., 2005; Warren et al., 2010, Vrticka, Bondolfi, et al. 2012), although the thus-far observed patterns are rather heterogeneous. Some studies report an under-recruitment of brain regions normally used to down-regulate emotional states associated with increased emotional behavioral and brain responses. Other investigations found stronger prefrontal cortex activation associated with reduced emotion regulation efficiency, or increased vulnerability to distraction by attachment-relevant emotional information entailing the requirement for greater cognitive control. However, these discrepancies can (at least partly) be explained by different task instructions, either requiring participants to actively and directly down-regulate emotional states, or to pay attention to task-relevant, non-emotional information while being exposed to task-irrelevant, emotional (attachment-related) stimuli. In the present study, participants were instructed to pay attention to and to attribute positive and negative adjectives to themselves (and a close other). Within such a context, the observed activation pattern of increased DLPFC activation may be indicative of reduced emotion regulation efficiency and/or increased vulnerability to task distraction. Such interpretation is in accordance with our account of amygdala activity likely reflecting increased relevance attribution to positive and negative self-representations.

Overall, the currently available fMRI literature on attachment anxiety corroborates the notion that adolescents with a negative attachment-derived self-model may: (i) attribute stronger relevance and implicit attention to self-attributes, (ii) employ diverse (semantic, social contextual, and memory-retrieval related) processes to embed their self-representations in a personally meaningful context, and (iii) show impaired cognitive control/emotion regulation capacities. Our findings importantly extend such characteristics relating to attachment anxiety by adding specificity through directly measuring the effects of an underlying negative self-model. Interestingly, our analyses reveal that a negative attachment-derived self-model appears to not only be associated with increased neural processing of negative evaluations of the self – likely because the latter sustain negative self-representations – as hypothesized, but also with heightened activity to positive self-evaluations. It therefore appears, at least in our adolescent population, that a negative self-model characterized by attachment anxiety may generally enhance the neural processing of self-attributes, regardless of valence. Future investigations are encouraged to investigate in more depth how the neural activity underlying self-representations relates to valence as a function of attachment anxiety and/or the attachment-derived self-model.

#### Self-model and brain activity during negative adjective attribution to the close other

Besides suggesting that a negative self-model is associated with increased brain activity during positive and negative trait-adjective evaluation related to the self, our fMRI data also provide preliminary evidence for an opposite relation between a negative self-model and a negative trait-adjective evaluation concerning a close other. We observed that activity in the left amygdala/parahippocampus and left DLPFC decreased during negative trait-adjective attribution to the close other as a function of adolescents’ negative self-model. Again, such activity overlapped with the main effect of task adjective attribution > syllable counting and thus suggests that a low positivity of self-concept is associated with differential recruitment of brain areas generally involved in adjective attribution when affected participants think about others in a negative way.

In terms of amygdala and parahippocampus activation (discussed above to reflect saliency processing and attachment-related memory retrieval), our findings seem to indicate that the initial relation between adolescents’ self-model and the contrast self > close other can be decomposed into two opposing patterns. Whereas the latter neural computations as a function of adolescents’ self-model seem to be more prominent during evaluation of adjectives concerning the self, these computations are less apparent during evaluation of negative adjectives concerning the close other. According to our previous interpretation, adolescents with a negative self-model may therefore attribute less relevance to internally derived negative close other attributes, and may less readily employ memory-retrieval-related processes to embed such negative close other representations in a personally and socially meaningful context.

With regard to left DLPFC activation, we have suggested above that a negative attachment-derived self-model may be indicative of reduced emotion regulation efficiency and/or increased vulnerability to task distraction during positive and negative adjective attribution to the self due to the higher relevance of such information. For negative adjective attribution to the close other, our data shows the exact opposite effect, which may suggest that adolescents’ emotion regulation during the evaluation of negative adjectives concerning the close other was more efficient, or more likely less required due to weaker task distraction. Such an interpretation is again corroborated by reduced amygdala activity during the negative close other condition, possibly reflecting decreased salience of negative-other adjectives.

The findings regarding brain activity during other-representation do not correspond to our primary hypothesis. Because the attachment-derived other-model (negatively) maps to the attachment avoidance dimension, we mainly expected to find decreased brain activity during positive adjective attribution during the close other condition as a function of the other-model. Instead, we observed decreased brain activity during negative adjective attribution to the close other as a function of the self-model. One potential explanation for such discrepancy may once more be that in our experiment, the other was the participants’ best (same sex) friend, which means that participants’ representation of the other may have been particularly positive, and thus less likely to be influenced by the negativity of the other-model. In fact, the behavioral data suggests that in our experiment, close other-representation was even more positive than self-representation. Associated with such a pattern may also have been a stronger variance in the link between self- and close other-representations and the attachment-derived self-model, potentially limiting the detection of any effects of the other-model on brain activity. As already discussed above, future investigations should look at the neural underpinnings of self- and other-representations as a function of attachment style and/or attachment-derived self- and other-models by including different other conditions along an extended other-closeness dimension.

#### Self-model and additional brain activations

Besides observing differential recruitment of brain activity during adjective attribution as a function of individual differences in the attachment-derived self model within areas showing a general involvement in adjective attribution (see above), we also found that one extensive cluster mainly encompassing the cerebellum (and extending to the fusiform face area [FFA] and pons) showed a similar activation pattern, despite only minimally overlapping with the main effect of task (i.e., adjective attribution > syllable counting). Activity in this particular cluster therefore appears to have been additionally recruited/selectively deactivated in adolescents with a high negativity of the self-model. Still, the activation pattern in this cluster strongly resembled the activation reported above, namely (i) increased neural processing of positive and negative self-attributes, but (ii) decreased neural processing of negative other-attributes. As for the (pre)cuneus, Gündel et al. (2003) have previously reported increased cerebellar activation in a study investigating grief through the exposure of bereaved women to pictures of their deceased loved one. Regarding the cerebellum, the latter association was understood as concomitant to a “feeling of being drawn toward” the stimulus, possibly reflecting the activation of automatic motor programs. More generally speaking, a recent meta-analysis of over 350 fMRI studies provided evidence for strong involvement of the cerebellum in social cognition, and particularly in processes with a high level of abstraction, including the description of behaviors in terms of traits (Van Overwalle, Baetens, Marien, & Vandekerckhove, 2014). Our findings pertaining to a negative self-model in adolescents could therefore be linked to similar processing known to occur in the cerebellum. What is concerning the FFA, activity in this area is prominently associated with face processing (Vuilleumier & Pourtois, 2007). In our study, participants with a negative self-model may thus have used visual imagery during adjective attribution – an interpretation that accords with our discussion of the effects we observed in the (pre)cuneus. Because the FFA was not found for the main effect of task (adjective attribution > syllable counting), participants with a negative self-model may have additionally recruited this area, beyond the usual memory-related imagery associated with adjective attribution observed in the (pre)cuneus across all participants.

#### Age and brain activity during adjective attribution

We have previously reported some age effects on brain activity during a different (“social feedback processing”) task in another sample of adolescents within a similar age range (Vrtička et al., 2014). Furthermore, another investigation (Pfeifer et al., 2013) found that activity in the MOFC/VMPFC during self-evaluations increased with age (as well as pubertal development) in adolescents aged 10–13 years, particularly when they were evaluating social (vs. academic) competence. Despite such previous findings of age-related effects, the present investigation did not reveal any significant influence of age on the neural correlates of adjective attribution, including in the MOFC/VMPFC. Possible explanations for such discrepancy may be the different age ranges investigated in our versus previous studies, in addition to the fact that we employed a cross-sectional design comprising only adolescents and not (young) adolescents versus adults (e.g., Pfeifer et al., 2009; Jankowski et al., 2014), which may have precluded us from capturing enough variance regarding age – as functional brain maturation is known to follow different, and often non-linear, paths in different brain areas, and to last beyond adolescence (Casey, Jones, & Hare, 2008). In addition, in our previous study (Vrtička et al., 2014), we used a rather different task, involving the processing of externally provided social information (vs. the internal representation of self- and other-attributes applied here), which may have been more susceptible to age influence. Future studies using an adolescent versus adult sample or, even better, employing a longitudinal design may reveal more information regarding possible age differences in adjective attribution during adolescence, particularly pertaining to the contrast self versus close other, as well as the time of emergence of such effects (early vs. late adolescence).

## Conclusion

Overall, our data on the putative neural substrates of self- and other-representations as a function of attachment in adolescents suggest that, when evaluating positive and negative adjectives regarding either the self or a close other, the attachment-derived self-model is associated with (i) increased neural processing of positive and negative self-attributes, but (ii) decreased neural processing of negative close other-attributes. These findings support the general view that an increased relevance of the internally generated self-view is an important underlying characteristic of the attachment anxiety dimension, and for the first time propose a neural basis that may be linked to this core feature of attachment anxiety, if reproduced by independent investigations. Furthermore, our results indicate that during adolescence, a negative-self view is also associated with a weaker allocation of mental resources during attribution of negative adjectives to a close other, possibly reflecting an idealization of the close other at the expense of a more nuanced perspective on his/her personality (Mikulincer & Shaver, 2007). Future studies would need to confirm this inclination of disregarding negative traits of close others as a function of a negative attachment-derived self-model, and also investigate its developmental course by either including an adult control population or following up the sample longitudinally. Future investigations more specifically assessing the impact of a negative attachment-derived self-model (in association with the attachment anxiety dimension) on social-emotional development during adolescence are indicated by the present results, and may help to integrate findings regarding cerebral and behavioral changes during adolescence.

## Electronic supplementary material

Supplemental Material DOCX 38.8 kb
