# Supplementary material for: Brain activity underlying negative self- and other-perception in adolescents: The role of attachment-derived self-representations
Source: Cogn Affect Behav Neurosci. 2017 Feb 6;17(3):554–76. doi: 10.3758/s13415-017-0497-9 (PMC5403860; doi:10.3758/s13415-017-0497-9)
Supplement: Supplementary file 1 — DOCX 38.8 kb [file 13415_2017_497_MOESM1_ESM.docx]

**Supplement:**

Please find below a complete list of used adjectives (taken from Anderson, 1968) for our version of the TAET.

| **List #** | **Positive words** | | **Anderson #** | **Likeableness** |
| --- | --- | --- | --- | --- |
|  | *English original* | *French Translation* |  |  |
| 1, 2, 3 | sincere | sincère | 3 | 573 |
| 1, 2, 3 | kind | gentil | 15 | 520 |
| 1, 2, 3 | dependable | fiable | 10 | 536 |
| 1, 2, 3 | warm | chaleureux | 14 | 522 |
| 1, 2, 3 | polite | poli | 30 | 489 |
| 1, 2, 3 | punctual | ponctuel | 43 | 466 |
| 1, 2, 3 | intelligent | intelligent | 9 | 537 |
| 1, 2, 3 | imaginative | imaginatif | 28 | 492 |
| 1, 2, 3 | confident | confiant | 70 | 401 |
| 1, 2, 3 | energetic | énergique | 52 | 457 |
|  |  |  |  |  |
| 1 | honest | honnête | 4 | 555 |
| 1 | outgoing | extraverti | 67 | 412 |
| 1 | truthful | droit | 7 | 545 |
| 1 | modest | modeste | 60 | 428 |
| 1 | thoughtful | sensé | 11 | 529 |
| 1 | understanding | compréhensif | 5 | 549 |
| 1 | friendly | sympa | 16 | 519 |
| 1 | calm | calme | 69 | 406 |
| 1 | practical | pragmatique | 62 | 425 |
| 1 | thrifty | économe | 78 | 372 |
| 1 | studious | studieux | 64 | 418 |
| 1 | observant | observateur | 41 | 467 |
| 1 | pleasant | plaisant | 25 | 495 |
| 1 | ambitious | ambitieux | 32 | 484 |
| 1 | careful | prudent | 72 | 390 |
|  |  |  |  |  |
| 2 | helpful | serviable | 27 | 492 |
| 2 | enthusiastic | enthousiaste | 29 | 489 |
| 2 | clever | ingénieux | 24 | 496 |
| 2 | attentive | attentif | 54 | 450 |
| 2 | amusing | amusant | 50 | 460 |
| 2 | witty | spirituel | 35 | 480 |
| 2 | cooperative | coopératif | 39 | 476 |
| 2 | idealistic | idéaliste | 74 | 384 |
| 2 | frank | franc | 55 | 450 |
| 2 | trustful | candide | 22 | 504 |
| 2 | happy | heureux | 17 | 514 |
| 2 | orderly | ordonné | 71 | 399 |
| 2 | inquisitive | questionneur | 65 | 413 |
| 2 | logical | logique | 44 | 465 |
| 2 | considerate | prévenant | 12 | 527 |
|  |  |  |  |  |
| 3 | generous | généreux | 51 | 459 |
| 3 | loyal | loyal | 6 | 547 |
| 3 | cheerful | gai | 21 | 504 |
| 3 | talented | doué | 37 | 478 |
| 3 | sociable | sociable | 59 | 429 |
| 3 | independent | indépendant | 53 | 455 |
| 3 | efficient | efficace | 33 | 482 |
| 3 | romantique | romantique | 79 | 371 |
| 3 | patient | patient | 36 | 478 |
| 3 | tolerant | tolérant | 49 | 461 |
| 3 | curious | curieux | 58 | 432 |
| 3 | neat | soigneux | 42 | 466 |
| 3 | humorous | comique | 19 | 505 |
| 3 | creative | créatif | 47 | 462 |
| 3 | responsible | responsable | 20 | 505 |

**Table 1: List of all used positive adjectives.** Adjectives are provided in their original (English) version as well as in their French translation. Adjectives are sorted according to their appearance in one (or all) of the three lists. Original item numbers as well as likeableness ratings (Anderson, 1968) are provided as well.

| **List #** | **Negative words** | | **Anderson #** | **Likeableness** |
| --- | --- | --- | --- | --- |
|  | *English original* | *French Translation* |  |  |
| 1, 2, 3 | jealous | jaloux | 31 | 104 |
| 1, 2, 3 | gossipy | ragoteur | 40 | 119 |
| 1, 2, 3 | cruel | cruel | 6 | 40 |
| 1, 2, 3 | loud-mouthed | grande-gueule | 19 | 83 |
| 1, 2, 3 | hot-tempered | colérique | 52 | 152 |
| 1, 2, 3 | wasteful | dépensier | 59 | 160 |
| 1, 2, 3 | lazy | paresseux | 43 | 126 |
| 1, 2, 3 | pessimistic | pessimiste | 63 | 164 |
| 1, 2, 3 | sloppy | brouillon | 54 | 153 |
| 1, 2, 3 | clumsy | maladroit | 82 | 199 |
|  |  |  |  |  |
| 1 | untrustworthy | traître | 11 | 65 |
| 1 | unsociable | asocial | 60 | 161 |
| 1 | phony | faux-jeton | 4 | 27 |
| 1 | conceited | prétentieux | 15 | 74 |
| 1 | inconsistent | incohérent | 75 | 193 |
| 1 | unattentive | insensible | 62 | 164 |
| 1 | mean | méchant | 5 | 37 |
| 1 | nervous | nerveux | 79 | 196 |
| 1 | noisy | bruyant | 68 | 173 |
| 1 | greedy | avare | 14 | 72 |
| 1 | disobedient | désobéissant | 45 | 128 |
| 1 | superstitious | superstitieux | 73 | 189 |
| 1 | impolite | impoli | 28 | 103 |
| 1 | envious | envieux | 57 | 157 |
| 1 | rude | bourru | 16 | 76 |
|  |  |  |  |  |
| 2 | insincere | hypocrite | 13 | 66 |
| 2 | ungrateful | ingrat | 34 | 109 |
| 2 | unintelligent | stupide | 66 | 168 |
| 2 | self-centered | égocentrique | 23 | 96 |
| 2 | cold | froid | 37 | 113 |
| 2 | discourteous | grossier | 35 | 110 |
| 2 | malicious | retors | 10 | 52 |
| 2 | stubborn | borné | 78 | 196 |
| 2 | liar | menteur | 3 | 26 |
| 2 | distrustful | méfiant | 25 | 99 |
| 2 | cowardly | peureux | 36 | 110 |
| 2 | unobservant | inattentif | 76 | 194 |
| 2 | fault-finding | pinailleur | 51 | 148 |
| 2 | moody | lunatique | 71 | 182 |
| 2 | obnoxious | odieux | 9 | 48 |
|  |  |  |  |  |
| 3 | egotistical | égoïste | 38 | 116 |
| 3 | dishonest | malhonnête | 7 | 41 |
| 3 | irritable | irritable | 49 | 143 |
| 3 | boring | ennuyant | 24 | 97 |
| 3 | ill-mannered | mufle | 22 | 95 |
| 3 | indifferent | indifférent | 83 | 202 |
| 3 | untidy | désordonné | 69 | 175 |
| 3 | oversensitive | hypersensible | 70 | 179 |
| 3 | dominating | dominateur | 55 | 153 |
| 3 | narrow-minded | obtus | 17 | 80 |
| 3 | nosey | indiscret | 27 | 102 |
| 3 | boastful | vantard | 41 | 122 |
| 3 | quarrelsome | querelleur | 26 | 101 |
| 3 | possessive | possessif | 72 | 183 |
| 3 | irresponsible | irresponsable | 33 | 106 |

**Table 2: List of all used negative adjectives.** Adjectives are provided in their original (English) version as well as in their French translation. Adjectives are sorted according to their appearance in one (or all) of the three lists. Original item numbers as well as likeableness ratings (Anderson, 1968) are provided as well.

**Reference:**

Anderson, N.H. (1968) Likableness ratings of 555 personality-trait words. J Pers Soc Psychol, 9:272-9.
